# Development of a GIN‐McMaster Checklist Extension for Out‐of‐Hospital Health Guidelines: A Research Protocol

**DOI:** 10.1002/gin2.70060

**Published:** 2026-01-28

**Authors:** Brendan V. Schultz, Timothy H. Barker, Emma Bosley, Amy Keir, Christian Martin‐Gill, Dannielle Pollock, Eddy Lang, Elie A. Akl, Holger J. Schünemann, Jennifer Petkovic, Joanne Khabsa, Louise Reynolds, Michael McCaul, Michelle Thomson, Miloslav Klugar, Paul Simpson, Robin Pap, Sonja Maria, Wojtek Wiercioch, Zachary Munn

**Affiliations:** ^1^ Faculty of Health and Medical Science, School of Public Health, Health Evidence Synthesis, Recommendations, and Impact (HESRI) Adelaide University Adelaide South Australia Australia; ^2^ Department of Health, Queensland Government Queensland Ambulance Service Brisbane Queensland Australia; ^3^ School of Clinical Sciences, Faculty of Health Queensland University of Technology Brisbane Queensland Australia; ^4^ SA Ambulance Service Eastwood South Australia Australia; ^5^ College of Medicine and Public Health Flinders University Bedford Park South Australia Australia; ^6^ School of Medicine University of Pittsburgh Pittsburgh Pennsylvania USA; ^7^ Emergency Medicine University of Calgary Calgary Alberta Canada; ^8^ Department of Internal Medicine American University of Beirut Beirut Lebanon; ^9^ Clinical Epidemiology and Research Center (CERC) Humanitas University Milan Italy; ^10^ Faculty of Medicine, Bruyere Research Institute University of Ottawa Ottawa Ontario Canada; ^11^ Clinical Research Institute, American University of Beirut Medical Center Beirut Lebanon; ^12^ Safer Care Victoria Melbourne Victoria Australia; ^13^ Faculty of Health Sciences, School of Nursing, Midwifery and Paramedicine Australian Catholic University Melbourne Victoria Australia; ^14^ Violet Vines Marshman Centre for Rural Health Research La Trobe University Bundoora Victoria Australia; ^15^ Division of Epidemiology and Biostatistics, Department of Global Health, Faculty of Medicine and Health Sciences Stellenbosch University Cape Town South Africa; ^16^ JBI, School of Public Health, Faculty of Health and Medical Sciences The University of Adelaide Adelaide South Australia Australia; ^17^ Cochrane Czech Republic, Czech Republic: A JBI Centre of Excellence, Czech GRADE Network, Institute of Health Information and Statistics of the Czech Republic Prague Czech Republic; ^18^ Faculty of Education, Center of Evidence‐based Education and Arts Therapies: A JBI Affiliated Group Palacký University Olomouc Olomouc Czech Republic; ^19^ School of Health Sciences Western Sydney University Sydney New South Wales Australia; ^20^ School of Nursing, Paramedicine and Healthcare Sciences Charles Sturt University Port Macquarie New South Wales Australia; ^21^ Department of Health Research Methods, Evidence, and Impact McMaster University Hamilton Ontario Canada; ^22^ Michael G. DeGroote Cochrane Canada & McMaster GRADE Centres McMaster University Hamilton Ontario Canada

**Keywords:** GIN‐McMaster checklist, GRADE, guideline development, guidelines, health guidelines, out‐of‐hospital, pre‐hospital

## Abstract

**Introduction:**

The delivery of healthcare in the out‐of‐hospital setting by paramedics, emergency medical technicians, and first responders is informed by various health guidelines that cover a myriad of medical presentations. These guidelines often score poorly on quality assessment tools and do not routinely meet accepted standards for guideline development. To address this, we propose the development of an extension to the Guideline International Network (GIN)‐McMaster Guideline Development Checklist (GDC) to assist developers of health guidelines on out‐of‐hospital care.

**Methods:**

The aim of this project are to: (i) identify the current methodologies, approaches and processes used by developers of guidelines on out‐of‐hospital care and determine how they align with the original GIN‐McMaster GDC; (ii) understand the overarching barriers and enablers of guideline development in this setting; (iii) generate a fit‐for‐purpose extension through an iterative consensus‐based approach; and (iv) disseminate this document to target users.

**Discussion:**

This protocol outlines the proposed development of an extension to the GIN‐McMaster GDC, designed specifically for developers of health guidelines on out‐of‐hospital care. The development of this extension will be informed by understanding current practices and the determinants that influence guideline development in this setting. This will involve reviewing all items within the original checklist to determine if modifications and/or new items are required.

## Summary

- Out‐of‐hospital health guidelines are commonly used by paramedics, emergency medical technicians and first responders.
- These guidelines do not routinely meet accepted standards for guideline development and score poorly on quality assessment tools.
- This protocol describes the intended methodology that will be used to create a contextualised out‐of‐hospital extension to the GIN–McMaster Guideline Development Checklist.
- To inform the development of this extension, we will first survey and interview developers of out‐of‐hospital guidelines to understand their current practices and the barriers and enablers that influence guideline enterprise in this setting.
- The extension will then be developed through an iterative consensus‐based approach which will involve pilot‐testing prior to finalisation.

## 1 Introduction

Health guidelines are ubiquitous in modern healthcare and are comprised of systematically developed statements that address clinical, health policy, or public health questions [1, 2]. These guidelines are commonly used by paramedics, emergency medical technicians and first responders to inform the emergency therapies provided to patients attended to in the out‐of‐hospital environment. The primary role of these clinicians has rapidly evolved in recent decades from facilitating transportation to hospital under the strict supervision of a physician to operating as autonomous healthcare practitioners in some geographical jurisdictions [3, 4, 5, 6]. This expansion in clinical scope has resulted in a proliferation of out‐of‐hospital guidelines being produced for managing the various conditions, maladies, and traumatic injuries patients in this setting may present with.

In our previous scoping review of guidelines in this field, we identified these documents often scored poorly on various appraisal instruments [7]. This review identified and aggregated 311 appraisals of out‐of‐hospital guidelines, and found the average AGREE‐II Domain 3 (‘rigour of development’) score was 55.6%. This is a concerning finding given the various manuals, frameworks, and software solutions that are currently available to aid developers of out‐of‐hospital health guidelines. These developers are often faced with creating guidelines using evidence that is heterogenous, indirect or imprecise which presents distinct obstacles to following common methodological approaches [8, 9]. Due to the inherent ethical, logistical and feasibility considerations associated with conducting research in the out‐of‐hospital setting, comparative evidence of treatment effects is seldom available to inform guideline development [10]. As shown in the context of rare diseases that face similar challenges, innovative approaches to evidence elicitation, aggregation and synthesis may be required to assist developers of out‐of‐hospital guidelines [11].

The Guideline International Network (GIN)‐McMaster Guideline Development Checklist (GDC) is widely acknowledged as a key resource for informing guideline development and is comprised of 146 unique items that should be considered when planning, developing, implementing, evaluating, and updating guidelines [12]. Unlike other tools that critique or evaluate credibility, the checklist is designed to function as a practical resource when planning and developing a health guideline. Since 2014, the GIN‐McMaster GDC has been formally ‘extended’ to guide quality assurance and improvement [13], rapid guidelines [14], interest‐holder engagement [15], and equity [16, 17], with extensions also planned for guideline adaptation and the use of artificial intelligence [18, 19]. While these extensions describe specific items and/or concepts that may also be considered during guideline development, no extension has been developed that contextualises the GDC for guideline developers in a specific area of medicine. We believe that operationalizing the generic nature of the checklist and nuancing the examples it contains will increase adherence to recommended practices and ultimately improve guideline quality in this field.

While we have previously identified that out‐of‐hospital guidelines are often appraised as being of medium to low‐quality [7], the granular elements of how these documents are specifically developed and implemented remain unclear. Previous literature has reported that these guidelines are predominately developed *de novo*, however, there has been limited exploration of the specific techniques, approaches, and processes currently used [20]. Similarly, the barriers and enablers that influence guideline development in this setting require further investigation. We believe by first determining the exact practices used by these guideline developers and eliciting the underpinning determinants that predispose these behaviours, a contextualized fit‐for‐purpose extension to the GIN‐McMaster GDC can be created. The objective of this extension is to improve the rigour, transparency and trustworthiness of guidelines developed to guide the provision of emergency healthcare in the out‐of‐hospital setting.

## 2 Methodology

**Figure 1:**
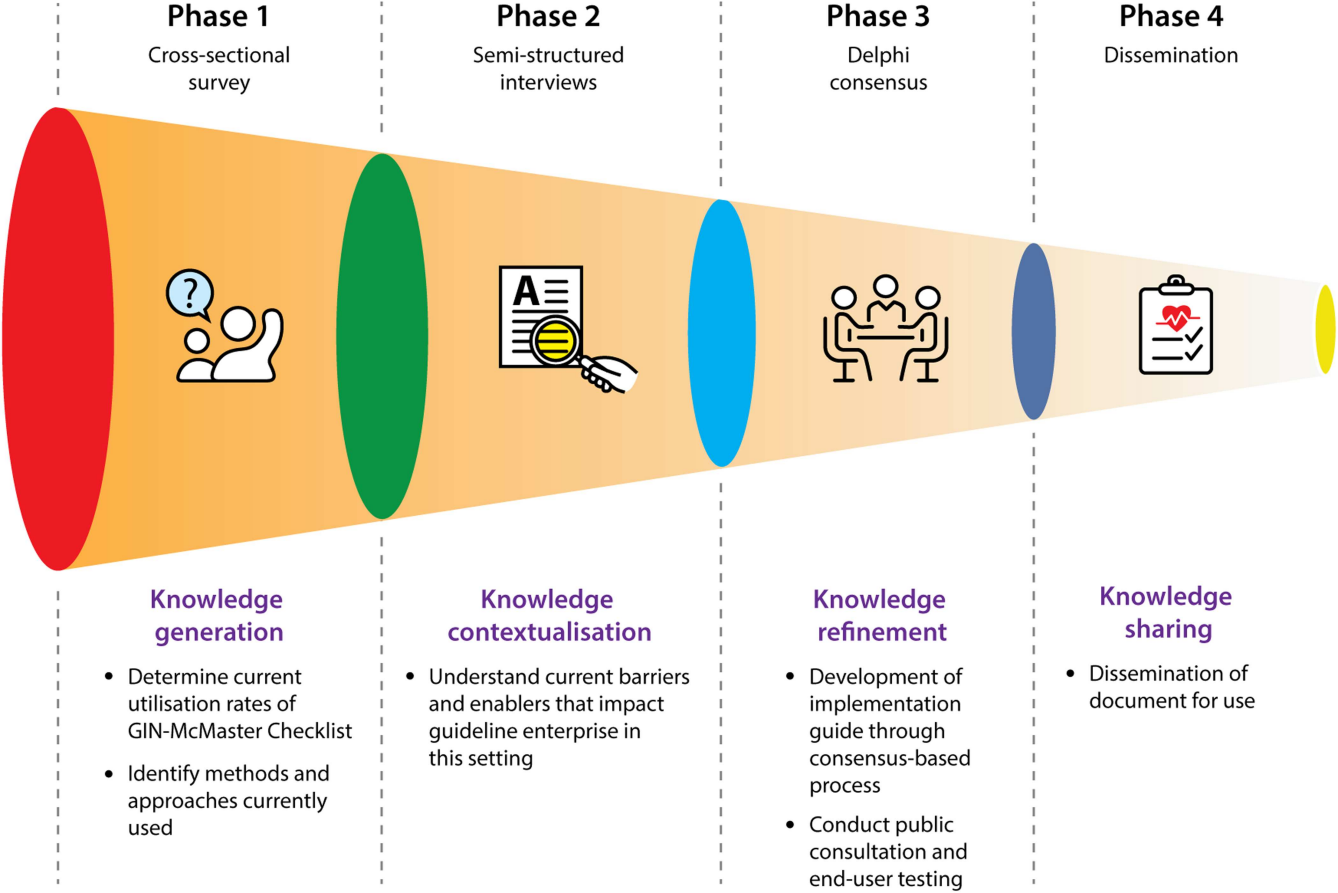
Planned research phases.

This project will comprise four discernible phases: Phase 1 – conduct a cross‐sectional survey to identify what methodologies and/or approaches are commonly used by developers of health guidelines on out‐of‐hospital care and determine how this practice aligns with GIN‐McMaster GDC; Phase 2 – perform semi‐structured interviews to identify the barriers and enablers that influence out‐of‐hospital guideline development; Phase 3 – generate the extension through a consensus modified Delphi approach and conduct appropriate interest‐holder engagement; and Phase 4 – disseminate this extension to target users (refer to Figure 1). While these phases are sequentially numbered, this research will be conducted iteratively with continual refinement. We aim to complete this work within 2 years.

In this project, we consider an out‐of‐hospital guideline to be a document that contains recommendations that address clinical, operational or system questions relevant to the delivery of healthcare in the out‐of‐hospital setting that are informed by the best available evidence, expert judgement, and interest‐holder input [1, 2, 21].

### 2.1 Project Working Groups

To facilitate the completion of this project, we will create an overarching steering committee and interest‐holder panel. All participants will be required to disclose any potential or actual conflicts of interest that may arise through any phase of this project, with this appropriately documented.

#### 2.1.1 Steering Committee

The steering committee will oversee and coordinate all phases of this research. This group will be comprised of methodologists with experience in guideline development and/or the GIN‐McMaster GDC, and expert developers of out‐of‐hospital guidelines. These individuals will be personally invited to participate based on their prior experiences. Members of this group will oversee the following items: (i) protocol generation; (ii) obtaining ethics approval for the cross‐sectional survey and interviews; (iii) developing survey questions and recruiting participants; (iv) conducting interviews; (v) generating the draft GIN‐McMaster extension; (vi) organizing and leading the Delphi process; and (vii) reporting all findings.

#### 2.1.2 Interest‐Holder Panel

To ensure the outcomes of this project are fit‐for‐purpose and can be utilised by developers of out‐of‐hospital guidelines, we will recruit a representative group of interest‐holders with legitimate interests in the topic under consideration [22]. This group will be comprised of expert developers of out‐of‐hospital guidelines (who represent prospective end‐users of the extension), guideline methodologists, paramedics, emergency medical technicians and first responders (who represent healthcare providers that commonly use these guidelines), and members of the public (who will be treated under guidelines informed by the extension). We will define expert developers of out‐of‐hospital guidelines as individuals who have produced equal to or greater than five out‐of‐hospital guidelines in their career. While expert out‐of‐hospital healthcare providers will be defined as clinicians with a minimum of ten years clinical experience who maintain clinical practice and demonstrate additional expertise through education, quality improvement activities or professional contributions to the field. These individuals will be identified and subsequently recruited through advertising of various platforms, including GIN connect, LinkedIn, various out‐of‐hospital forums, as well as by suggestion of members of the steering committee. Where possible, we will aim to balance this group based on gender and geographical location. The primary function of this group will be involvement in the Delphi consensus process and providing comments, consultation, and recommendations on preliminary drafts of the GIN‐McMaster GDC extension.

### 2.2 Phase 1: Determining the Methods Currently Used by Out‐of‐Hospital Guideline Developers

In our previously published scoping review, we identified that out‐of‐hospital guidelines have poor methodological rigour and are of low to medium quality when assessed against structured appraisal instruments [7]. This exercise detailed the current state of guidelines in this setting, though did not determine the exact methods and/or approaches developers of out‐of‐hospital guidelines use and if these processes align with steps contained within the GIN‐McMaster GDC. To address this, we plan to conduct an online anonymous cross‐sectional survey of developers of out‐of‐hospital guidelines using Qualtrics (Provo, UT).

To identify potential respondents, we will utilise a non‐random (snowballing strategy) to recruit the target population (developers of out‐of‐hospital guidelines). To ensure the sample population is representative of developers globally, and to mitigate publication and geographic bias we intend to use a multifaceted recruitment approach. First, the survey and participant information sheet will be emailed to the corresponding author of out‐of‐hospital guidelines included in our previous scoping review and guidelines listed in the Prehospital Guidelines Consortium Registry [7, 23]. These authors will be encouraged to share the survey link with other guideline developers in their region that do not routinely publish in academic journals. Second, we will proactively contact various ambulance agencies, emergency medical services and professional medical associations that are known to produce out‐of‐hospital guidelines requesting distribution of the survey through their organisation mailing lists or professional networks. Third, the survey will be shared through the social media platform LinkedIn to maximise international reach.

Given there is currently no accepted population denominator for developers of out‐of‐hospital guidelines globally, an *a priori* sample size cannot be calculated. Prior to dissemination, the survey will be piloted and pre‐tested to minimise measurement error and ensure the language used is correctly interpreted. Ethical approval for this survey has been approved by the University of Adelaide Faculty of Health and Medical Sciences Lower Risk Human Research Ethics Committee (H‐2025‐004; January 15th 2025).

Upon receiving an invitation to complete the survey, respondents will be required to confirm that they have previously been involved in the development of an out‐of‐hospital guideline. If respondents indicate they meet this criteria, they will be asked to complete the remaining questions in the survey. The survey will consist of two sections. The first will capture demographic information about the respondents such as the country they reside in, how many guidelines they have previously developed, and if they have completed formal training in guideline development (e.g. INGUIDE). The second component will be comprised of binary questions (yes/no) that will ask respondents to reflect on how their current practice aligns with the GIN‐McMaster GDC. These questions will then branch into free‐text responses where respondents will be asked to provide further information on the methods, approaches, or techniques they currently use and provide case examples where possible. Survey questions will be informed by the 146 items and 18 overarching topics contained within the GIN‐McMaster GDC. The proposed list of survey questions are displayed in Supporting Information 1.

Following data collection, we will then perform a basic qualitative content analysis, whereby these responses will be grouped into categories based on the observed similarities and differences [24]. These results will then be presented in both tabular and diagrammatic formats, with an accompanying narrative that will be reported in alignment with the Checklist for Reporting Survey Studies (CROSS) [25].

### 2.3 Phase 2: Understanding Barriers and Enablers That Influence Out‐of‐Hospital Guideline Development

To ensure the extension is nuanced and tailored for developers of out‐of‐hospital guidelines, we will conduct a qualitative enquiry to elicit the barriers and enablers that influence guideline development in this setting. We will recruit developers of out‐of‐hospital guidelines from the participants involved in the earlier cross‐sectional survey. Data will be collected via one‐on‐one, semi‐structured interviews conducted in an online format using Zoom (Zoom Video Communications, CA) or a similar digital adjunct as required. Prior to commencement, ethical approval will be sought through the University of Adelaide Faculty of Health and Medical Sciences Lower Risk Human Research Ethics Committee.

We intend to recruit 15 participants, with this number selected as the likely number required to reach information redundancy or data saturation when analysis and interpretation are performed [26]. These interviews will be recorded and subsequently transcribed verbatim (orthographically) using the auto‐transcription function within Zoom. To ensure uniformity, interviews will be undertaken by one researcher, using an *a priori* question guide that will be piloted tested prior to use. The draft interview guide is displayed in Supporting Material 1. To minimise interviewer bias, we will utilise peer‐checking whereby a second researcher will observe a selection of interviews, and member‐checking whereby participants will be provided a copy of the interview transcription prior to analysis to verify its accuracy.

Following data collection, we will perform a content analysis using a combination of inductive and deductive coding based on principles outlined by Elo and Kyngas [27]. The GIN‐McMaster GDC will be used as an *a priori* framework for analysis and the organisation of data into categories. To facilitate analysis, raw data will first be entered into NVivo (Lumivero, CO) with coding and subsequent category generation performed independently by two researchers to increase validity [28]. Overall, analysis will be undertaken using inductive and deductive techniques with emerging categories from the collected data mapped against the GIN‐McMaster GDC. The positionality of each member of the research team will be transparently described, and reflexivity practiced throughout by way of collaborative discussion about the data, analytic process, the emerging categories, and where the researchers are located within it.

We will narratively describe the dominant categories and associated sub‐categories identified through this analysis, and then link them to the 18 topics of the GIN‐McMaster GDC as appropriate. This analysis will include the verbatim use of quotations as supporting evidence to describe the identified barriers and enablers of guideline development in the out‐of‐hospital setting. The findings of this phase of the project will be reported in alignment with the Consolidated Criteria for Reporting Qualitative Research (COREQ) checklist [29].

### 2.4 Phase 3: Development of Extension Through a Consensus‐Based Approach

Informed by the knowledge generated in the prior phases, we will then conduct a multi‐stage consensus process to develop an out‐of‐hospital extension for the GIN‐McMaster GDC. This will be performed using a modified e‐Delphi methodology with the panel provided a set of questions to consider rather than the traditional Delphi method which commences with open‐ended questions to elicit ideas and discussion [30]. This will be reported in accordance with the Accurate Consensus Reporting Document (ACCORD) checklist [31]. Prior to commencing this work, ethical approval will be sought through the University of Adelaide Faculty of Health and Medical Sciences Lower Risk Human Research Ethics Committee.

We intend to recruit a representative panel of 20–40 interest‐holders to ensure the sample size is sufficient to provide an acceptable level of replicability [32]. We will aim for this panel to have the following composition: (i) 50% out‐of‐hospital guideline developers; (ii) 20% guideline methodologists; (iii) 20% out‐of‐hospital healthcare providers; (iv) 10% members of the public. It is envisaged that a proportion of the panel will be comprised of individuals who may have participated in earlier study phases. To ensure members of the public can effectively contribute in panel discussions, the steering committee will provide specific education sessions as required.

Following panel recruitment, the steering committee will convene to ensure all members are familiar with the original checklist and to review findings of the previous phases. The committee will then review all 146 items contained in the original checklist and preliminary draft the out‐of‐hospital extension based on the findings from the prior phases. Informed by the methodology proposed to develop the GIN‐McMaster GDC adaptation extension [18], items in this extension will be classified as either: (i) modified from original checklist; (ii) new item not contained in the original checklist but pertinent for out‐of‐hospital guideline development; (iii) original item from checklist that is unchanged; and (iv) item that may be deemed non‐applicable by end‐users. In instances that an item is deemed to require modification, the steering committee will draft the proposed wording.

Using Qualtrics (Provo, UT), we will then create multiple web‐based questionnaires that will ask the Delphi panel to answer on a seven‐point Likert scale (1 = strongly disagree, 7 = strongly agree) if they support the ‘classification’ that has been assigned to each step [30]. In the first round, respondents will also be provided a free‐text box to suggest changes to wording that has been proposed for items requiring modification. These will be considered by three members of the steering committee and incorporated iteratively if deemed appropriate. The consensus threshold for inclusion will be defined as 80% agreement amongst respondents (≥ 80% responding ‘somewhat agree’, ‘agree’ or ‘strong agree’) [33, 34, 35]. Following each round of the Delphi, item classifications that do not reach this threshold will be discussed and iteratively modified by the steering committee until consensus is reached for each item [18]. To supplement the items contained in the extension, the steering committee will also develop the following: (a) a detailed explanation of each step; (b) associated out‐of‐hospital specific exemplars (where available); and (c) operational guidance on how to implement the extension item. This will be presented to the Delphi panel in a series of online meetings for endorsement.

All decisions made regarding this process will be documented and narratively described as appropriate. The first draft of the completed extension will then be made publicly available for consultation with active feedback sought from other interest‐holders that have not been involved in the Delphi to ensure validity of the document. This version of the extension will then be provided to out‐of‐hospital guideline development groups for pilot testing in a real‐world context, with these groups invited to provide feedback on its usability, function and format. All suggested amendments will be reviewed and considered by the steering committee before the document is submitted to a representative of McMaster University and the GIN board for endorsement and finalisation.

### 2.5 Phase 4: Dissemination of Extension

To ensure this extension is accessible to all developers of out‐of‐hospital health guidelines, we intend to publish the finalised document in an open‐access journal, and make it available on the GIN‐McMaster checklist website. Following publication, this will be prospectively circulated with all individuals who have been involved in any capacity with this work so it can be incorporated and/or adopted into their practice. Similarly, to augment dissemination, we plan to present the extension at various out‐of‐hospital conferences, forums, and workshops.

## 3 Discussion

In this protocol we outline the development of a GIN‐McMaster GDC extension that is contextualized for developers of out‐of‐hospital guidelines. Developed through a consensus process, this document will detail items that should be considered for guideline development in the out‐of‐hospital setting. This work will be informed by first quantifying the current practices of developers and then understanding the determinants of guideline enterprise in this environment.

### 3.1 Implications for Clinical Practice

The purpose of developing this extension is to improve the rigour and overall trustworthiness of guidelines used by paramedics, emergency medical technicians, and first responders in the out‐of‐hospital setting. It is well acknowledged that poorly developed guidelines represent a risk to patient safety as they may recommend ineffective treatments [36, 37]. The development of this extension should directly address this issue by standardizing the process of producing these guidelines. This may also have tangible downstream effects in reducing evidence waste in this field by ensuring more high‐quality guidelines are produced which can then be adopted and/or adapted for local contexts. Additionally, this work may result in future collaboration between developers who produce guidelines for the out‐of‐hospitalsetting through relationship building and networking.

### 3.2 Strength and Limitations

This work is strengthened by the rigorous development methodology we have outlined, which will ensure the validity of the extension that is produced. Rather than building a novel instrument from scratch, we have made the deliberate decision to build upon the GIN‐McMaster GDC, which is an existing high‐quality and validated adjunct. This will ensure that information provided to guideline developers is reliable and reflects current best practice. We believe by first quantifying current practices and determining factors that influence this behaviour, this document will be fit‐for‐purpose and easily adoptable into current practices.

This protocol should be read with acknowledgement of the following limitations. Namely, the potential methodological issues regarding the use of a modified e‐Delphi technique, which relies on the selection of appropriate participants. As an emerging medical field, it may be difficult to quantify what constitutes an expert in this field. We believe this shortcoming will be mitigated by the interest‐holder consultation we plan to perform.

## Author Contributions

**Brendan V. Schultz:** conceptualization, methodology, project administration, writing – original draft. **Timothy H. Barker:** conceptualization, methodology, supervision, writing – review and editing. **Emma Bosley:** conceptualization, methodology, supervision, writing – review and editing **Amy Keir:** writing – review and editing. **Christian Martin‐Gill:** writing – review and editing. **Dannielle Pollock:** writing – review and editing. **Eddy Lang:** writing – review and editing. **Elie A. Akl:** writing – review and editing. **Holger J. Schünemann:** writing – review and editing. **Jennifer Petkovic:** writing – review and editing. **Joanne Khabsa:** writing – review and editing. **Louise Reynolds:** writing – review and editing. **Michael McCaul:** writing – review and editing. **Michelle Thomson:** writing – review and editing. **Miloslav Klugar:** writing – review and editing. **Paul Simpson:** writing – review and editing. **Robin Pap:** writing – review and editing: **Sonja Maria:** writing – review and editing. **Wojtek Wiercioch:** writing – review and editing. **Zachary Munn:** conceptualization, methodology, supervision, writing – review and editing.

## Ethics Statement

As this study is a protocol for future research, the authors have nothing to report.

## Conflicts of Interest

Prof. Zachary Munn, Prof. Holger J. Schünemann, Dr Miloslav Klugar and Dr Wojtek Wiercioch currently hold positions on the Editorial Board of Clinical and Public Health Guidelines but did not participate in the editorial process of this article nor influence editorial decisions related to it. The remaining authors declare no conflict of interest relevant to this research.

## Supporting information

Supplementary Material 1.

## Data Availability

Data sharing not applicable – no new data generated, or the article describes entirely theoretical research.
