# Supplementary material for: Development of a GIN‐McMaster Checklist Extension for Out‐of‐Hospital Health Guidelines: A Research Protocol
Source: Clin Public Health Guidel. 2026 Jan 28;3(2):e70060. doi: 10.1002/gin2.70060 (PMC13563315; doi:10.1002/gin2.70060)
Supplement: Supplementary file 1 [file GIN2-3-e70060-s001.docx]

**Development of a GIN-McMaster checklist extension for out-of-hospital health guidelines: a research protocol – Supplementary Material 1**

A. Proposed list of survey questions

Determining the methodology used to develop out-of-hospital health guidelines

Start of Block: Consent Form

In the out-of-hospital setting, the delivery of healthcare by paramedics, emergency medical technicians, and first-responders is informed by overarching health guidelines (also known as ‘clinical practice guidelines’ or ‘CPGs’). This research aims to understand the current methodologies and approaches used to create these guidelines by surveying guideline developers. It is anticipated the survey will take approximately 15-30 minutes to complete, with your responses captured in a non-identifiable format.

The research is undertaken by Mr Brendan Schultz, a PhD candidate with the University of Adelaide under the supervisor of Professor Zachary Munn, Dr Timothy Barker, and Dr Emma Bosley. By clicking the button below, you acknowledge:

1. I agree to take part in this research project which has been approved by the University of Adelaide Human Research Ethics Committee (approval number: H-2025-004).
2. I have had the project, so far as it affects me, and the potential risks and burdens fully explained to my satisfaction by the research team.
3. I have had the opportunity to ask any questions I may have about the project and my participation. My consent is given freely.
4. Although I understand the purpose of the research project is to improve the quality of health/medical care, it has also been explained that my involvement may not be of any benefit to me.
5. I agree to participate in the activities outlined in the participant information sheet I understand that as my participation is anonymous and I can withdraw any time up until submission of the survey.
6. I understand that if I decide not to take part or withdraws from the project, that this will not affect medical advice in the management of my health, now or in the future.
7. I have been informed that the information gained in the project may be published in a journal article and/or presented at medical conferences.
8. I have been informed that in the published materials I will not be identified and any personal information that could identify me will not be divulged.
9. I consent for the use of my data by the same or other researchers in future research projects that are in the same general area of research (for example, genealogical, ethnographical, epidemiological, or chronic illness research).
10. I understand personal information that may identify me (e.g. name, address, date of birth) will be removed or changed before it is shared with other researchers.
11. I understand my information will only be disclosed according to the consent provided, except where disclosure is required by law.
12. I am aware that I should keep a copy of this Consent Form, when completed, and the relevant Information Sheet. This can occur by requesting a copy of your responses once completing this survey.

- I consent, begin the study (1)
- I do not consent, I do not wish to participate (2)

End of Block: Consent Form

Start of Block: Demographic Questions

Q0 We would like to know a little information about you

Q1 Have you developed an out-of-hospital (also known as ‘prehospital’) guideline?

- Yes (1)
- No (2)

Skip To: End of Survey If Have you developed an out-of-hospital or prehospital guideline? = No

Q2 What is your current professional occupation? (tick all that apply)

- Paramedic / EMT (1)
- Physician (2)
- Academic (3)
- Guideline Methodologist (4)
- Guideline Developer (5)
- Other (please specify) (6) __________________________________________________

Q3 What country you predominately work in?

________________________________________________________________

| Page Break |  |
| --- | --- |

Q4 Have you undertaken formal training in guideline development?

- Yes (1)
- No (2)

Display this question:

If Have you undertaken formal training in guideline development? = Yes

Q4 (A) Please describe this training

________________________________________________________________

________________________________________________________________

________________________________________________________________

________________________________________________________________

________________________________________________________________

Q5 Have you undertaken formal training in systematic reviews or evidence synthesis?

- Yes (1)
- No (2)

Display this question:

If Have you undertaken formal training in systematic reviews or evidence synthesis? = Yes

Q5 (A) Please describe this training

________________________________________________________________

________________________________________________________________

________________________________________________________________

________________________________________________________________

________________________________________________________________

End of Block: Demographic Questions

Start of Block: Main Survey

Q6 **In your career**, how many guidelines have you been involved in?

________________________________________________________________

Q7 **In the past 12 months**, how many guidelines have you been involved in?

________________________________________________________________

| Page Break |  |
| --- | --- |

Q8 Which group best describes the organisation you have **predominately** developed guidelines with?

- Individual ambulance service or EMS agency (1)
- National EMS group (e.g. JRCALC, NASEMSO, NAEMSP) (2)
- Professional medical society (e.g. Stroke Foundation, EAST) (3)
- Academic group (e.g. Group of academics with no clear affiliation) (4)
- National institute or governing body (e.g. NICE, SIGN, AHA, ILCOR) (5)
- Other (please specify) (6) __________________________________________________

| Page Break |  |
| --- | --- |

Q9 Have you heard of the GIN-McMaster Guideline Development Checklist?

- Yes (1)
- No (2)

Display this question:

If Have you heard of the GIN-McMaster Guideline Development Checklist? = Yes

Q9A Have you previously used the GIN-McMaster Guideline Development Checklist when developing a guideline?

- Yes (1)
- No (2)

| Page Break |  |
| --- | --- |

Q The following questions will ask you about specific elements of guideline development. Please answer these questions based on **your predominant experiences** when developing guidelines that are used by paramedics, emergency medical technicians and first-responders.

| Page Break |  |
| --- | --- |

Q10 In the guidelines you have been involved in, was an overarching guideline development group and/or committee formed?

- Yes (1)
- No (2)

Display this question:

If In the Guidelines you have been involved in, was an overarching guideline development group and/or comm... = No

Q10 A What process (if any) was used instead?

________________________________________________________________

________________________________________________________________

________________________________________________________________

________________________________________________________________

________________________________________________________________

Display this question:

If In the Guidelines you have been involved in, was an overarching guideline development group and/or comm... = Yes

Q11 What types of individuals were involved in these development groups? (tick all that apply)

- Consumers (patients or carers) (1)
- End-users (clinicians who will use the GUIDELINE in clinical practice) (2)
- Methodology experts (3)
- Health economists (4)
- Content experts (5)
- Health executives or decision-makers (6)
- Epidemiologists (7)
- Other (please specify) (8) __________________________________________________

Display this question:

If In the Guidelines you have been involved in, was an overarching guideline development group and/or comm... = Yes

Q12 How did you identify which interest-holders ('stakeholders') should be involved/consulted with during the guideline development process?

________________________________________________________________

________________________________________________________________

________________________________________________________________

________________________________________________________________

________________________________________________________________

Display this question:

If In the Guidelines you have been involved in, was an overarching guideline development group and/or comm... = Yes

Q13 What process was used to recruit or enroll these individuals to join the development group?

________________________________________________________________

________________________________________________________________

________________________________________________________________

________________________________________________________________

________________________________________________________________

Display this question:

If In the Guidelines you have been involved in, was an overarching guideline development group and/or comm... = Yes

Q14 How was the size of the development group determined?

________________________________________________________________

________________________________________________________________

________________________________________________________________

________________________________________________________________

________________________________________________________________

Display this question:

If In the Guidelines you have been involved in, was an overarching guideline development group and/or comm... = Yes

Q15 How were internal disagreements or disputes among group members managed (if they occurred)?

________________________________________________________________

________________________________________________________________

________________________________________________________________

________________________________________________________________

________________________________________________________________

Display this question:

If In the Guidelines you have been involved in, was an overarching guideline development group and/or comm... = Yes

Q16 Do you have any case examples demonstrating the methods that were used to form a guideline development group? (this can be a DOI link or name of the guideline)

________________________________________________________________

________________________________________________________________

________________________________________________________________

________________________________________________________________

________________________________________________________________

Display this question:

If If Do you have any case examples demonstrating the methods that were used to form a guideline develo... Text Response Is Displayed

Q Alternatively, you can upload a file below

| Page Break |  |
| --- | --- |

Q17 Was a budget created that outlined the estimated costs of guideline development?

- Yes (1)
- No (2)

Display this question:

If Was a budget created that outlined the estimated costs of GUIDELINE development? = No

Q17 A What process (if any) was used instead?

________________________________________________________________

________________________________________________________________

________________________________________________________________

________________________________________________________________

________________________________________________________________

Q18 Have you previously applied for specific grant funding to support guideline development?

- Yes (1)
- No (2)

| Page Break |  |
| --- | --- |

Q19 In the guidelines you have been involved in, were individuals provided specific training or education?

- Yes (1)
- No (2)

Display this question:

If In the Guidelines you have been involved in, were individuals provided specific training or education? = Yes

Q19 A Please describe the training provided

________________________________________________________________

________________________________________________________________

________________________________________________________________

________________________________________________________________

________________________________________________________________

| Page Break |  |
| --- | --- |

Q20 In the guidelines you have been involved in, were individuals required to complete a conflict-of-interest (COI) declaration?

- Yes (1)
- No (2)

Display this question:

If In the Guidelines you have been involved in, were individuals required to complete a conflict-of-intere... = No

Q20 B What process (if any) was used instead?

________________________________________________________________

________________________________________________________________

________________________________________________________________

________________________________________________________________

________________________________________________________________

Display this question:

If In the Guidelines you have been involved in, were individuals required to complete a conflict-of-intere... = Yes

Q21 Was the conflict-of-interest declaration made publicly available when the guideline was disseminated?

- Yes (1)
- No (2)

Display this question:

If In the Guidelines you have been involved in, were individuals required to complete a conflict-of-intere... = Yes

Q22 A Was an overarching conflict-of-interest policy developed and/or utilised?

- Yes (1)
- No (2)

Display this question:

If Was an overarching conflict-of-interest policy developed and/or utilised? = Yes

Q If available, please upload a copy of this policy below

| Page Break |  |
| --- | --- |

Q22 In the guidelines you have been involved in, was a scoping document or protocol created that outlined the objectives of the guideline, estimated timeline for completion and intended methodology?

- Yes (1)
- No (2)

Display this question:

If In the Guidelines you have been involved in, was a scoping document or protocol created that outlined t... = Yes

Q22 A What information was contained in this document?

________________________________________________________________

________________________________________________________________

________________________________________________________________

________________________________________________________________

________________________________________________________________

Display this question:

If In the Guidelines you have been involved in, was a scoping document or protocol created that outlined t... = No

Q22 B What process (if any) was used instead?

________________________________________________________________

________________________________________________________________

________________________________________________________________

________________________________________________________________

________________________________________________________________

| Page Break |  |
| --- | --- |

Q21 In the guidelines you have been involved in, was a priority setting exercise undertaken to determine the key topics that will be covered?

- Yes (1)
- No (2)

Display this question:

If In the Guidelines you have been involved in, was a priority setting exercise undertaken to determine th... = No

Q21 A What process (if any) was used instead?

________________________________________________________________

________________________________________________________________

________________________________________________________________

________________________________________________________________

________________________________________________________________

Display this question:

If In the Guidelines you have been involved in, was a priority setting exercise undertaken to determine th... = Yes

Q21 B What did this involve?

________________________________________________________________

________________________________________________________________

________________________________________________________________

________________________________________________________________

________________________________________________________________

Display this question:

If In the Guidelines you have been involved in, was a priority setting exercise undertaken to determine th... = Yes

Q22 Do you have any case examples demonstrating priority setting exercises that have been performed? (this can be a DOI link or name of the guideline)

________________________________________________________________

________________________________________________________________

________________________________________________________________

________________________________________________________________

________________________________________________________________

Display this question:

If If Do you have any case examples demonstrating priority setting exercises that have been performed? (this can be a DOI link or name of the GUIDELINE) Text Response Is Displayed

Q Alternatively, you can upload a file below

| Page Break |  |
| --- | --- |

Q23 In the guidelines you have been involved in, were specific research questions created that the guideline would address?

- Yes (1)
- No (2)

Display this question:

If In the Guidelines you have been involved in, were specific research questions created that the guidelin... = No

Q23 A What process (if any) was used instead?

________________________________________________________________

________________________________________________________________

________________________________________________________________

________________________________________________________________

________________________________________________________________

Display this question:

If In the Guidelines you have been involved in, were specific research questions created that the guidelin... = Yes

Q24 Were these questions structured using a specific format? (ie PICO, PICOT, PEO, SPIDER)?

- Yes (1)
- No (2)

Display this question:

If Were these questions structured using a specific format? (ie PICO, PICOT, PEO, SPIDER)? = No

Q25 A What format was used instead?

________________________________________________________________

________________________________________________________________

________________________________________________________________

________________________________________________________________

________________________________________________________________

Display this question:

If Were these questions structured using a specific format? (ie PICO, PICOT, PEO, SPIDER)? = Yes

Q26 How were the outcome(s) of the research questions determined? ie used of a ranking system or voting process

________________________________________________________________

________________________________________________________________

________________________________________________________________

________________________________________________________________

________________________________________________________________

| Page Break |  |
| --- | --- |

Q27 In the guidelines you have been involved in, was a research protocol created that described the methodology that would be used to identify, select, and synthesise evidence?

- Yes (1)
- No (2)

Display this question:

If In the Guidelines you have been involved in, was a research protocol created that described the methodo... = Yes

Q27 A What was included in this protocol?

________________________________________________________________

________________________________________________________________

________________________________________________________________

________________________________________________________________

________________________________________________________________

Display this question:

If In the Guidelines you have been involved in, was a research protocol created that described the methodo... = No

Q27 B What process (if any) was used instead?

________________________________________________________________

________________________________________________________________

________________________________________________________________

________________________________________________________________

________________________________________________________________

| Page Break |  |
| --- | --- |

Q28 In the guidelines you have been involved in, what overarching methodologies have been used to create recommendations? (tick all that apply)

- De novo development (creation of new recommendations) (1)
- Adoption (using recommendations contained in existing guidelines with minimal	changes) (2)
- Adaption (modifying or altering recommendations contained in existing guidelines for local use) (3)
- Adolopment (mixture of adoption, adaption and de novo methods) (4)
- Other (please specify) (5) __________________________________________________

Display this question:

If In the Guidelines you have been involved in, what overarching methodologies have been used to create re... = De novo development (creation of new recommendations)

Q29 A What did **de novo development** involve? (tick all that apply)

- Searching for existing systematic reviews (4)
- Searching grey literature (5)
- Conducting your own systematic review or rapid review (6)
- Other (please specify) (7) __________________________________________________

Display this question:

If In the Guidelines you have been involved in, what overarching methodologies have been used to create re... = Adoption (using recommendations contained in existing Guidelines with minimal changes)

Q29 B Do you have any case examples demonstrating when **adoption** **methods** have been used?  (this can be a DOI link or name of the guideline)

________________________________________________________________

________________________________________________________________

________________________________________________________________

________________________________________________________________

________________________________________________________________

Display this question:

If If Do you have any case examples demonstrating when adoption methods have been used?  (this can be a... Text Response Is Displayed

Q Alternatively, you can upload a file below

Display this question:

If In the Guidelines you have been involved in, what overarching methodologies have been used to create re... = Adaption (modifying or altering recommendations contained in existing Guidelines for local use)

Q29 C Do you have any case examples demonstrating when **adaption methods** have been used? (this can be a DOI link or name of the guideline)

________________________________________________________________

________________________________________________________________

________________________________________________________________

________________________________________________________________

________________________________________________________________

Display this question:

If If Do you have any case examples demonstrating when adaption methods have been used? (this can be a... Text Response Is Displayed

Q Alternatively, you can upload a file below

Display this question:

If In the Guidelines you have been involved in, what overarching methodologies have been used to create re... = Adolopment (mixture of adoption, adaption and de novo methods)

Q30 Do you have any case examples demonstrating when **adolopment methods** have been used? (this can be a DOI link or name of the guideline you have developed)

________________________________________________________________

________________________________________________________________

________________________________________________________________

________________________________________________________________

________________________________________________________________

Display this question:

If If Do you have any case examples demonstrating when adolopment methods have been used? (this can be... Text Response Is Displayed

Q Alternatively, you can upload a file below

Display this question:

If What did de novo development involve? (tick all that apply) = Searching for existing systematic reviews

Q30 A - 1 Were the systematic reviews that were identified appraised using a validated tool? (ie AMSTAR, ROBIS)

- Yes (1)
- No (2)

| Page Break |  |
| --- | --- |

Q31 In the guidelines you have been involved in, was evidence summarised into as an evidence to decision table, evidence profile or summary of findings table?

- Yes (1)
- No (2)

Display this question:

If In the Guidelines you have been involved in, was evidence summarised into as an evidence to decision ta... = Yes

Q31 A Do you have any case examples demonstrating evidence tables, evidence profiles or summary of evidence tables? (this can be a DOI link or name of the guideline)

________________________________________________________________

________________________________________________________________

________________________________________________________________

________________________________________________________________

________________________________________________________________

Display this question:

If If Do you have any case examples demonstrating evidence tables, evidence profiles or summary of evid... Text Response Is Displayed

Q Alternatively, you can upload a file below

Display this question:

If In the Guidelines you have been involved in, was evidence summarised into as an evidence to decision ta... = No

Q31 A What process (if any) was used instead?

________________________________________________________________

________________________________________________________________

________________________________________________________________

________________________________________________________________

________________________________________________________________

| Page Break |  |
| --- | --- |

Q32 In the guidelines you have been involved in, was a specific framework used to access the certainty of evidence? (e.g. GRADE or USPSTF)

- Yes (1)
- No (2)

Display this question:

If In the Guidelines you have been involved in, was a specific framework used to access the certainty of e... = Yes

Q32 A Please specify the framework you have used

________________________________________________________________

________________________________________________________________

________________________________________________________________

________________________________________________________________

________________________________________________________________

Display this question:

If In the Guidelines you have been involved in, was a specific framework used to access the certainty of e... = No

Q32 B What process (if any) was used instead?

________________________________________________________________

________________________________________________________________

________________________________________________________________

________________________________________________________________

________________________________________________________________

| Page Break |  |
| --- | --- |

Q33 In the guidelines you have been involved in, what information was considered when making a recommendation? (tick all that apply)

- Health benefits and harms (1)
- Certainty in the available evidence (2)
- Estimated costs (3)
- Feasibility (4)
- Acceptability (5)
- Equity (6)
- Values and preferences (7)
- Other (please specify) (8) __________________________________________________

| Page Break |  |
| --- | --- |

Q34 When developing recommendations, was the strength of the recommendation included? (ie 'weak' or 'strong')

- Yes (1)
- No (2)

Display this question:

If When developing recommendations, was the strength of the recommendation included? (ie 'weak' or '... = No

Q34 A What process (if any) was used instead?

________________________________________________________________

________________________________________________________________

________________________________________________________________

________________________________________________________________

________________________________________________________________

Q35 When developing recommendations, was the quality of evidence included? (ie high, moderate, low, very low)

- Yes (1)
- No (2)

Display this question:

If When developing recommendations, was the quality of evidence included? (ie high, moderate, low, v... = No

Q35 A What process (if any) was used instead?

________________________________________________________________

________________________________________________________________

________________________________________________________________

________________________________________________________________

________________________________________________________________

| Page Break |  |
| --- | --- |

Q36 In the guidelines you have been involved in, was a summary outlining the rationale for each recommendation provided?

- Yes (1)
- No (2)

Display this question:

If In the Guidelines you have been involved in, was a summary outlining the rationale for each recommendat... = No

Q36 A What process (if any) was used instead?

________________________________________________________________

________________________________________________________________

________________________________________________________________

________________________________________________________________

________________________________________________________________

Q37 In the guidelines you have been involved in, how were recommendations agreed upon? (tick all that apply)

- Delphi method (1)
- Group voting (2)
- Other (please specify) (3) __________________________________________________

| Page Break |  |
| --- | --- |

Q38 In the guidelines you have been involved in, was standardised wording used when creating recommendations?

- Yes (1)
- No (2)

Q Do you have any case examples demonstrating how recommendations were generated, developed or worded? (this can be a DOI link or name of the guideline)

________________________________________________________________

________________________________________________________________

________________________________________________________________

________________________________________________________________

________________________________________________________________

Display this question:

If If Do you have any case examples demonstrating how recommendations were generated, developed or worded? (this can be a DOI link or name of the GUIDELINE) Text Response Is Displayed

Q Alternatively, you can upload a file below

| Page Break |  |
| --- | --- |

Q39 In the guidelines you have been involved in, was the guideline made available for peer-review or public comments prior to dissemination?

- Yes (1)
- No (2)

Display this question:

If In the Guidelines you have been involved in, was the guideline made available for peer-review or public... = No

Q39 A What process (if any) was used instead?

________________________________________________________________

________________________________________________________________

________________________________________________________________

________________________________________________________________

________________________________________________________________

Q40 In the guidelines you have been involved in, was the guideline pilot tested with end-users prior to implementation?

- Yes (1)
- No (2)

Display this question:

If In the Guidelines you have been involved in, was the guideline pilot tested with end-users prior to imp... = Yes

Q40 A What did this involve?

________________________________________________________________

________________________________________________________________

________________________________________________________________

________________________________________________________________

________________________________________________________________

| Page Break |  |
| --- | --- |

Q41 How were the guidelines you have been involved in disseminated?

________________________________________________________________

________________________________________________________________

________________________________________________________________

________________________________________________________________

________________________________________________________________

| Page Break |  |
| --- | --- |

Q42 In the guidelines you have been involved in, was auditing and/or monitoring conducted to see how recommendations were applied in clinical practice?

- Yes (1)
- No (2)

Display this question:

If In the Guidelines you have been involved in, was auditing and/or monitoring conducted to see how recomm... = Yes

Q Do you have any case examples demonstrating audit or monitoring tools that have been used? (this can be a DOI link or name of the guideline)

________________________________________________________________

________________________________________________________________

________________________________________________________________

________________________________________________________________

________________________________________________________________

Display this question:

If If Do you have any case examples demonstrating audit or monitoring tools that have been used? (this... Text Response Is Displayed

Q124 Alternatively, you can upload a file below

| Page Break |  |
| --- | --- |

Q43  In the guidelines you have been involved in, were tools provided to prospectively evaluate the effectiveness of the guideline after implementation?

- Yes (1)
- No (2)

Display this question:

If In the Guidelines you have been involved in, were tools provided to prospectively evaluate the effectiv... = Yes

Q43 A Do you have any case examples demonstrating this? (this can be a DOI link or name of the guideline)

________________________________________________________________

________________________________________________________________

________________________________________________________________

________________________________________________________________

________________________________________________________________

Display this question:

If If Do you have any case examples demonstrating this? (this can be a DOI link or name of the GUIDELINE) Text Response Is Displayed

Q Alternatively, you can upload a file below

| Page Break |  |
| --- | --- |

Q44 In the guidelines you have been involved in, were performance measures or quality indicators created?

- Yes (1)
- No (2)

Display this question:

If In the Guidelines you have been involved in, were performance measures or quality indicators created? = Yes

Q Do you have any case examples demonstrating this? (this can be a DOI link or name of the guideline)

________________________________________________________________

________________________________________________________________

________________________________________________________________

________________________________________________________________

________________________________________________________________

Display this question:

If If Do you have any case examples demonstrating this? (this can be a DOI link or name of the GUIDELINE) Text Response Is Displayed

QAlternatively, you can upload a file below

| Page Break |  |
| --- | --- |

Q44 In the guidelines you have been involved in, was a set policy, procedure or timeline used to determine when the guideline needs to be updated?

- Yes (1)
- No (2)

Display this question:

If In the Guidelines you have been involved in, was a set policy, procedure or timeline used to determine... = No

Q44 A What process (if any) was used instead?

________________________________________________________________

________________________________________________________________

________________________________________________________________

________________________________________________________________

________________________________________________________________

Display this question:

If In the Guidelines you have been involved in, was a set policy, procedure or timeline used to determine... = Yes

Q45 How was it determined if a partial or full update was required?

________________________________________________________________

________________________________________________________________

________________________________________________________________

________________________________________________________________

________________________________________________________________

Display this question:

If In the Guidelines you have been involved in, was a set policy, procedure or timeline used to determine... = Yes

Q Do you have any case examples demonstrating the methods or processes that have been used to update guidelines? (this can be a DOI link or name of the guideline)

________________________________________________________________

Display this question:

If If Do you have any case examples demonstrating the methods or processes that have been used to update Guidelines? (this can be a DOI link or name of the GUIDELINE) Text Response Is Displayed

Q Alternatively, you can upload a file below

| Page Break |  |
| --- | --- |

Q46 The next phase of this research involves conducting focus interviews to better understand the barriers and facilitators of out-of-hospital guideline development. If you would like to be involved, please enter your email address below.  Please note, this response is not linked or identifiable to any other questions in this survey.

________________________________________________________________

________________________________________________________________

________________________________________________________________

________________________________________________________________

________________________________________________________________

End of Block: Main Survey

B. Proposed interview guide

**Understanding the barriers and enablers that influence the development of out-of-hospital health guidelines**

## Demographic Questions

1. How would you describe your current professional occupation?

- *Prompt:* a) paramedic/EMT/first responder; b) physician; c) academic; d) guideline methodologist; e) guideline developer

2. How many out-of-hospital health guidelines have you developed in your career?

- *Prompt:* How many have you developed in the last 12 months?

3. Have you developed guidelines for more than one type of organisation? If so, could you describe them?

- *Prompt:* a) ambulance service/EMS agency; b) professional medical society; c) academic group; d) national institute or governing body

## Guideline Development Specific Questions

4. In your experience, what do you perceive are key elements that result in development of a high-quality out-of-hospital guideline?

- *Prompt:* If required to stimulate a response provide examples of potential elements: a) education/capability; b) resources/cost; c) lack of direct evidence create treatment recommendations; d) organisational constraints.
- *Prompt:* If multiple elements listed by participant; ask individual to rank/prioritise these – ‘what do you think is the biggest determinant’?
- *Prompt:* If required, consider asking participant is listed elements apply across all guidelines, or are limited to specific clinical topics/scope.
- *Prompt:* If required, consider asking participant if they can provide real-life examples or share personal experiences.

5. In your experience, what prevents or hinders out-of-hospital guideline development?

- *Prompt:* If required to stimulate a response provide examples of potential elements: a) education/capability; b) resources/cost; c) lack of direct evidence create treatment recommendations; d) organisational constraints.
- *Prompt:* If multiple elements listed by participant; ask individual to rank/prioritise these – ‘what do you think is the biggest obstacle to developing a high-quality guideline’?
- *Prompt:* If required, consider asking participant is listed elements apply across all guidelines, or are limited to specific clinical topics/scope.
- *Prompt:* If required, consider asking participant if they can provide real-life examples or share personal experiences.
